# Nivel de actividad física después de la implementación de un programa de ejercicio físico en escolares

**DOI:** 10.15446/rsap.V25n4.97374

**Published:** 2023-07-01

**Authors:** Nohora E. Álvarez-Rey, Lisette K. Cárdenas-Sandoval, William E. Atehortúa-Alarcón, Viannys S. Lamprea-Flórez

**Affiliations:** 1 NA: FT. Esp. Gerencia y Auditoría de la Calidad en Salud. Facultad de Ciencias Médicas y de la Salud, Universidad de Santander. Cúcuta, Colombia. no.alvarez@mail.udes.edu.co Universidad de Santander Facultad de Ciencias Médicas y de la Salud Universidad de Santander Cúcuta Colombia no.alvarez@mail.udes.edu.co; 2 LC: F T. Esp. Administración de la Salud. Facultad de Ciencias Médicas y de la Salud, Universidad de Santander. Cúcuta, Colombia. lis.cardenas@mail.udes.edu.co Universidad de Santander Facultad de Ciencias Médicas y de la Salud Universidad de Santander Cúcuta Colombia lis.cardenas@mail.udes.edu.co; 3 WA: Admin. Servicios de Salud. Esp. Estadística Aplicada. Facultad de Ciencias Médicas y de la Salud. Universidad de Santander, Cúcuta. wil.atehortua@mail.udes.edu.co Universidad de Santander Facultad de Ciencias Médicas y de la Salud Universidad de Santander Cúcuta wil.atehortua@mail.udes.edu.co; 4 VL: FT. Esp. Salud Ocupacional. Facultad de Ciencias Médicas y de la Salud, Universidad de Santander. Cúcuta, Colombia. vi.lamprea@mail.udes.edu.co Universidad de Santander Salud Ocupacional Facultad de Ciencias Médicas y de la Salud Universidad de Santander Cúcuta Colombia vi.lamprea@mail.udes.edu.co

**Keywords:** Ejercicio físico, actividad motora, adolescente, antropometría, programas *(fuente: DeCS, BIREME)*, Exercise, motor activity, adolescent, anthropometry, programs *(source: MeSH, NLM)*

## Abstract

**Objetivo:**

Relacionar medidas antropométricas y el nivel de actividad física pre y post implementación de un programa de ejercicio físico dirigido a escolares entre 11 y 14 años de una institución educativa pública.

**Métodos:**

Estudio experimental, la muestra estuvo conformada por 282 escolares, [211] grupo de intervención y [71] grupo control. Se realizó la aplicación del cuestionario PAQ-A (Physical Activity Questionnaire for Adolescent) y se tomaron las medidas antropométricas (peso, talla, perímetro abdominal, índice de masa corporal). Los investigadores diseñaron e implementaron un programa de ejercicio físico durante las clases de educación física.

**Resultados:**

En la valoración inicial el 28% de los estudiantes del grupo intervención clasificó como activo, proporción que se mantuvo para la valoración final sin cambios significativos (27,5%); el 28,1% del grupo control fue activo en la valoración inicial, proporción que ascendió a 31,2%, igualmente sin cambios significativos (p>0,05). Respecto al indicador IMC/edad, la proporción de estudiantes con sobrepeso u obesidad disminuyó del 40,3% en la pre intervención, al 37,6% en la post intervención.

**Conclusiones:**

El nivel de actividad física activo predominó en el género masculino, sin diferencias significativas, asimismo, se puede afirmar que decrece con la edad. El programa de ejercicio físico no tuvo efectos significativos en las medidas antropométricas de la población objeto de estudio, de forma indirecta se logró sensibilizar a los diferentes actores sobre la importancia de la práctica regular de la actividad física como factor protector de la salud.

## Abstract (en)

**Objective:**

To relate anthropometric measures and the level of physical activity pre and post implementation of a physical exercise program aimed at school children between 11 and 14 years of age in a public educational institution.

**Methods:**

Experimental study, the sample was made up of 282 school children, (211) intervention group and (71) control group. The PAQ-A (Physical Activity Questionnaire for Adolescents) was applied and anthropometric measurements were taken (weight, height, abdominal perimeter, body mass index). The researchers designed and implemented a physical exercise program during physical education classes.

**Results:**

At the initial assessment, 28% of the students in the intervention group were classified as active, a proportion that was maintained for the final assessment without significant changes (27.5%); 28.1% of the control group was active at the initial assessment, a proportion that rose to 31.2%, also without significant changes (p>0.05). Regarding the BMI/age indicator, the proportion of students with overweight or obesity decreased from 40.3% in the pre-intervention to 37.6% in the post-intervention.

**Conclusions:**

The level of active physical activity predominated in the male gender, without significant differences; likewise, it can be stated that it decreases with age. The physical exercise program did not have significant effects on anthropometric measures of the population under study, and indirectly it was possible to raise awareness among the different actors about the importance of regular practice of physical activity as a protective factor of health.

Según la Organización Mundial de la Salud (OMS), el sedentarismo constituye uno de los grandes desencadenantes y problemas en salud en los últimos tiempos 1; la inactividad física es el cuarto factor de riesgo de mortalidad más importante, el cual influye en la prevalencia de enfermedades no transmisibles (ENT). El macroestudio de escala global realizado en el 2018 por la OMS refiere que por causa de las ENT mueren 41 millones de personas cada año (71%). Las enfermedades cardiovasculares constituyen la mayoría de las muertes por ENT (17,9 millones cada año), seguidas del cáncer (9 millones), las enfermedades respiratorias (3,9 millones) y la diabetes (1,6 millones) 2.

En Colombia, según la Encuesta Nacional de Situación Nutricional (ENSIN) 3 realizada en el 2015, tres de cada diez niños y dos de cada diez niñas de tres a cinco años practican juego activo; y el exceso de peso aumentó de 4,9% en 2010 a 6,3% en 2015; sin embargo, en el país es más baja la situación con respecto a Centroamérica (7,4%) y a Suramérica (7,0%).

En edad escolar, el tiempo excesivo frente a pantallas, incluye ver televisión o jugar videojuegos, y afecta a siete de cada diez escolares de áreas urbanas, frente a cinco de cada diez de zonas rurales. El problema es más marcado entre la población de mayores ingresos, afectando a ocho de cada diez menores; y el exceso de peso en los menores se incrementó de 18,8% en 2010 a 24,4% en 2015. "La disminución de los niveles de actividad física (NAF), constituye uno de los factores de mortalidad más importantes en la actualidad a nivel mundial, y sin duda representa un detonante para el aumento de la posibilidad de padecer diferentes alteraciones de salud" 4, como las enfermedades cardiovasculares, la diabetes tipo 2 y la obesidad.

En el 2017, se llevó a cabo un proyecto donde se evaluó el NAF de los escolares en tres instituciones educativas públicas del municipio de Los Patios (Norte se Santander). Por medio del *Physical Activity Questionnaire for Adolescents* (PAQ-C), se evidenció que en el rol escolar se presentó un mayor índice de inactividad física ocasionado por las jornadas académicas extensas; otro factor influyente identificado fue la poca frecuencia y duración de las clases de educación física, debido a que se realizaban una vez a la semana con una duración de 2 horas; lo anteriormente expuesto comprueba que no se cumplen las recomendaciones de la OMS 5, que ponen el énfasis en que los niños y los jóvenes de 5 a 17 años deben invertir como mínimo 60 minutos diarios en actividades físicas de intensidad moderada a vigorosa.

Ante la problemática anteriormente expuesta y frente a los múltiples beneficios que se pueden lograr realizando actividad física (AF) desde temprana edad, se considera fundamental el diseño y la ejecución de programas de ejercicio físico en instituciones educativas, con base en evidencia científica, con el propósito de incentivar y concientizar a todos los actores sobre la importancia de generar una cultura de autocuidado y aprovechamiento de los espacios académicos, para favorecer el desarrollo psicosocial, las destrezas motoras y el aumento del NAF en el rol escolar, lo que genera cambios positivos en la condición de salud de la población.

Este estudio tiene como objetivo relacionar las medidas antropométricas y el nivel de actividad física pre y post implementación de un programa de ejercicio físico (PEF) dirigido a escolares entre 11 y 14 años de una institución educativa pública.

## MÉTODOS

Estudio con diseño de intervención, de tipo experimental, realizado con adolescentes de una institución educativa pública, asignados a un grupo de intervención (GI) y un grupo control (GC). Inicialmente, en el mes de agosto de 2019 se aplicó el cuestionario PAQ-A y se hizo la toma de medidas antropométricas (talla, peso, perímetro abdominal e índice de masa corporal (IMC)) pre y post intervención, realizada a través de un PEF que tuvo una duración de 16 semanas.

La población objeto de estudio fueron escolares de 11 a 14 años, matriculados en el 2019 y que cursaban los grados sexto, séptimo, octavo y noveno de una institución educativa pública. El estudio fue realizado en el censo de la población [282], el GI estuvo conformado por 211 participantes y el GC por 71; los criterios de inclusión fueron la aprobación del consentimiento y asentimiento informado, adolescentes de 11 a 14 años matriculados en el 2019 en la institución educativa; escolares con patologías osteomusculares fueron excluidos del estudio.

Para la recolección de la información se realizó el diligenciamiento del consentimiento y asentimiento informado, se caracterizó sociodemográficamente a la población mediante una encuesta. Después de ello, se aplicó el cuestionario PAQ-A al GI y al GC para determinar el NAF de los adolescentes pre y post intervención; este cuestionario evalúa el NAF y la frecuencia en los últimos 7 días durante el tiempo libre, entorno escolar y hogar. El puntaje final se obtiene de promediar las 8 primeras preguntas, la pregunta 9 identifica si se presentó alguna situación que impidió realizar AF. El diligenciamiento tiene una duración de 10-15 minutos; cada pregunta tiene 5 opciones de respuesta, las cuales están registradas en escala ordinal, cuyo equivalente es: 1(inactivo), 2 (poco activo), 3 (moderadamente activo), 4 (muy activo) y 5 (extremadamente activo) 6.

La toma de medidas antropométricas (talla, peso, IMC y perímetro abdominal) se llevó a cabo al GI y al GC pre y post intervención, basados en la Resolución 2465 del 2016, en la cual se adoptan los indicadores antropométricos, los patrones de referencia y los puntos de corte para la clasificación antropométrica del estado nutricional de niñas, niños y adolescentes menores de 18 años, adultos de 18 a 64 años y gestantes adultas 7. La talla se midió con un tallímetro, los indicadores y los puntos de corte utilizados fueron: > -1 talla adecuada para la edad, > -2 a < -1 riesgo de retraso en talla, y < -2 talla baja para la edad o retraso en talla.

Para medir el peso se utilizó una báscula digital, se registró el peso exacto en kilogramos. Posteriormente, se realizaron dos tomas para validar la medida; en los casos en que los valores fueron diferentes, se hizo una tercera toma, y se promediaron los dos valores más cercanos. Con respecto al IMC se tuvieron en cuenta los siguientes indicadores y puntos de corte: obesidad > +2, sobrepeso > +1 a < +2, IMC adecuado para la edad > -1 a < +1, riesgo de delgadez >-2 a < -1 y delgadez < -2.

Para finalizar se midió el perímetro abdominal y se registró en centímetros. Una vez recolectada la información, se diligenció en una base de datos para su posterior análisis.

Con base en la evidencia científica sobre programas de ejercicio físico y según las recomendaciones de la OMS que buscan mejorar los niveles de actividad física de los escolares, los investigadores diseñaron un PEF implementado en la población objeto de estudio una vez a la semana durante las clases de educación física, en los meses de agosto a noviembre de 2019.

El diseño del programa incluyó las dimensiones de frecuencia, intensidad, duración y tipo de ejercicio; en cuanto a la frecuencia, es importante resaltar que se realizó una vez a la semana, debido al horario asignado a la clase de educación física, el cual corresponde a dos horas. De acuerdo con el convenio establecido con la institución, el programa se aplicó durante una hora, y en la hora siguiente los estudiantes asistían a su clase convencional. La programación de los ejercicios se llevó a cabo de acuerdo con las cualidades físicas básicas del movimiento, las cuales son: flexibilidad, coordinación, equilibrio, velocidad, fuerza, resistencia, agilidad y destreza, es decir, ejercicios de tipo aeróbico, fortalecimiento muscular y óseo, con una intensidad de moderada a vigorosa.

El programa tuvo una duración de 60 minutos distribuidos en cinco fases: calentamiento (10 minutos), estiramiento inicial (10 minutos), fase activa (20 minutos), estiramiento final (10 minutos) y enfriamiento (10 minutos).

El análisis estadístico de la información se basó en la elaboración de distribuciones de frecuencia simple de las características sociodemográficas de los estudiantes; en ambas mediciones se calcularon medidas descriptivas para las variables antropométricas. El contraste de hipótesis para la comparación de los cambios observados entre las mediciones de los indicadores antropométricos y del NAF, se realizó mediante la prueba de rangos de Wilcoxon, en tanto que el contraste de hipótesis para establecer la asociación entre las características sociodemográficas y los indicadores antropométricos con el NAF, se realizó mediante la prueba de Chi cuadrado. Los cambios observados en el perímetro abdominal post intervención entre estudiantes activos e inactivos se evaluaron mediante la prueba U de Mann Whitney. El nivel de significancia establecido fue 0,05. El paquete estadístico utilizado fue SPSS versión 24.

El estudio fue presentado y aprobado por el Comité de Bioética de la Universidad de Santander, de acuerdo con la Resolución 8430 de 1993 del Ministerio de Salud, por la cual se establecen las normas científicas, técnicas y administrativas para la investigación en salud; esta investigación es de riesgo mínimo; asimismo, los procedimientos respetaron normas éticas concordantes con la Declaración de Helsinki. En la ejecución del proyecto prevaleció el criterio de respeto a la dignidad y la protección de los derechos de los escolares; se solicitó el consentimiento informado de los padres y el asentimiento informado de los niños y adolescentes.

## RESULTADOS

La población objeto de estudio fueron 282 escolares, de los cuales, el 59% [166] pertenecían al género femenino, y el 41% [116] al masculino; el 31% tenía 11 años, seguido del 26% con 12 años, el 25% con 14 años y el 18% con 13 años. Respecto al estrato socioeconómico, los escolares se encontraban en mayor representación en el estrato 2 con un 62%, seguido del estrato 3 con un 27%, y el estrato 1 con un 5%. En relación con el nivel de escolaridad de la madre, se encontró que el 53% completó la secundaria, el 22% eran universitarias y el 14% estudió una tecnología.

Se evidenció que las medidas descriptivas para el NAF en mediciones pre y post, en el GI el promedio fue 2,62, con desviación estándar 0,73 en la valoración inicial, y para la segunda medición el promedio se ubicó en 2,58, con desviación estándar 0,75. En el GC el promedio fue 2,56, con desviación estándar 0,77 en la pre intervención, y un promedio de 2,63, con desviación estándar 0,77, en post intervención.

De acuerdo con los resultados, en la valoración inicial el 28% de los estudiantes del GI clasificó como activo, proporción que se mantuvo para la valoración final sin cambios significativos (27,5%); en el GC el 28,1% fue activo en la valoración inicial, proporción que ascendió a 31,2%, igualmente sin cambios significativos (p > 0,05).

Al comparar los resultados de AF post intervención según variables sociodemográficas, solo se observaron diferencias estadísticamente significativas respecto a la edad (p=0,039), identificándose una mayor proporción de estudiantes activos en la edad de 11 años (37,9%). Las variables sexo, estrato y nivel de escolaridad de la madre no evidenciaron diferencias significativas (p>0,05) (Tabla 1).

**Tabla 1:** Clasificación del nivel de actividad física post intervención según características demográficas

Variable	Categoría	N	Clasificación del nivel de actividad física - post		X2	Valor p
			Activo	Inactivo		
Sexo	Masculino	87	30(34,5)	57(65,5)	3,52	0,062
	Femenino	131	30(22,9)	101(77,1)		
Edad	11	87	33(37,9)	54(62,1)	8,36	0,039
	12	47	10(21,3)	37(78,7)		
	13	28	7(25,0)	21(75,0)		
	14	56	10(17,9)	46(82,1)		
Estrato	Bajo	149	35(23,5)	114(76,5)	3,84	0,050
	Medio	69	25(36,2)	44(63,8)		
Nivel de escolaridad de la madre	Primaria	11	3(27,3)	8(72,7)	7,88	0,096
	Secundaria	115	25(21,7)	90(78,3)		
	Tecnología	31	9(29,0)	22(71,0)		
	Universidad	47	15(31,9)	32(68,1)		
	Otros	14	8(57,1)	6(42,9)		

X^2^ Chi Cuadrado.

Se hizo una comparación de los indicadores antropométricos pre y post del GI. Se observó un cambio estadísticamente significativo respecto al indicador talla/edad (p<0,05), observándose que en la valoración inicial solo el 36,7% tenía una talla adecuada para la edad, proporción que ascendió al 93,6% en la valoración final. Respecto al indicador iMc/edad, no se observaron cambios significativos, aunque la proporción con sobrepeso u obesidad disminuyó del 40,3% en la valoración inicial al 37,6% en la valoración final (Tabla 2).

**Tabla 2:** Comparativo de indicadores talla/edad e IMC/edad en grupo intervención

Indicador	Categoría	Medición		Prueba de Wilcoxon (Valor p)
		Antes n (%)	Después n (%)	
Talla / edad	Talla adecuada para la edad	80(36,7)	204(93,6)	0,000
	Riesgo de retraso en talla	133(61,0)	13(6,0)	
	Talla baja para la edad	5(2,3)	1(0,5)	
imc / edad	Delgadez	5(2,3)	6(2,8)	0,524
	Riesgo de delgadez	16(7,3)	17(7,8)	
	Adecuado para la edad	109(50,0)	113(51,8)	
	Sobrepeso	62(28,4)	53(24,3)	
	Obesidad	26(11,9)	29(13,3)	

Se comparó el NAF del GI con los indicadores antropométricos, y no se evidenciaron diferencias significativas (p>0,05) (Tabla 3).

**Tabla 3:** Nivel de actividad física post intervención del grupo intervención según indicadores antropométricos

Nivel de actividad física - post					
Categoría	N	Activo N (%)	Inactivo N (%)	X2	Valor p
Talla adecuada para la edad	204	56(27,5)	148(72,5)	2,71	0,257
Riesgo de retraso en talla	13	3(23,1)	10(76,9)		
Talla baja para la edad	1	1(100,0)	0(0,0)		
Delgadez	6	2(33,3)	4(66,7)	4,49	0,344
Riesgo de delgadez	17	3(17,6)	14(82,4)		
Adecuado para la edad	113	35(31,0)	78(69,0)		
Sobrepeso	53	10(18,9)	43(81,1)		
Obesidad	29	10(34,5)	19(65,5)		

X^2^ Chi Cuadrado.

En ambas mediciones se evaluó el comportamiento del perímetro abdominal de los estudiantes. En el GI el promedio de perímetro abdominal en la valoración inicial fue 69,1 cm, con desviación estándar de 8,6 cm; en la valoración final el promedio fue 69,5 cm, con desviación típica de 8,9 cm. En el GC el perímetro abdominal medio fue 69,7 cm, con desviación estándar de 8,5 cm en la primera medición; en la medición final, el promedio ascendió a 71,1 cm, con desviación típica de 8,9 cm. El rango para perímetro abdominal osciló entre 52 y 104 cm.

El promedio del perímetro abdominal del GI respecto al NAF fue menor en los estudiantes activos en comparación con los escolares inactivos, pero sin diferencias estadísticamente significativas (p>0,05).

## DISCUSIÓN

Se determinó el NAF pre y post implementación de un PEF dirigido a escolares entre 11 y 14 años de una institución educativa pública. De acuerdo con los resultados, no se evidenciaron cambios significativos en el GI ni en el GC, sin embargo, en el GC la proporción de nivel activo ascendió de 28,1 a 31,2% (p>0,05).

El PEF se implementó una vez a la semana, con una duración de 60 minutos, intensidad de moderada a vigorosa, con ejercicios de tipo aeróbico y anaeróbico, y la duración de la intervención fueron 16 semanas; estudios con objetivos similares a esta investigación reportan en relación con la efectividad de los programas, que no se presentan cambios significativos entre los GI 8-15. Otros estudios refieren que la aplicación de los programas debe complementarse con otras estrategias en el dominio escolar, hogar y tiempo libre para evidenciar efectos positivos en variables como IMC y perímetro abdominal, entre otras. En contraste con los resultados mencionados, algunos estudios afirman que se presentan cambios en las variables antropométricas y en los NAF post intervención, con duración de 60 minutos diarios acompañados de práctica deportiva 16-17, o 90 minutos diarios por 24 semanas 18.

De acuerdo con lo mencionado anteriormente, en este estudio no hubo aumento del NAF, es decir, el 28% de los escolares del GI era activo en la pre y en la post intervención; igualmente, un estudio realizado en cinco ciudades de Colombia reportó un bajo NAF de estudiantes, solamente el 15% realizaba AF durante 7 días a la semana y el 20% la practicaba apenas 5 veces, con una duración por sesión de 60 minutos 19. Asimismo, una investigación en la que participaron 89 alumnos de sexto de primaria distribuidos en GI y GC, se aplicó un PEF 5 veces a la semana durante 3 meses con intensidad moderada; posteriormente, se evalúo NAF con el cuestionario PAQ-A, el cual no reportó cambios con relación a la pre intervención 15.

Es importante resaltar que los resultados encontrados en este estudio en relación con el NAF pre y post intervención están influenciados por diferentes factores externos como la práctica de AF en los dominios hogar, tiempo libre e incluso el escolar, porque la institución educativa únicamente permite realizar la intervención con cada grupo de estudiantes una vez a la semana durante 60 minutos, lo cual no cumple con las recomendaciones de la OMS para mejorar la calidad de vida de los escolares y prevenir ENT. Además, se observó que en el entorno escolar no se implementan estrategias para promover la actividad física como factor protector de la salud, es decir, los estudiantes permanecen en el aula durante la jornada académica; en el recreo o descanso, un gran porcentaje de los escolares es inactivo, y pocos practican algún deporte como futbol o básquet. Otro determinante es el currículo escolar, el cual solo incluye dos horas semanales de clase de educación física 19-22.

A diferencia de los estudios anteriores, algunas investigaciones reportan aumento de la AF de moderada a vigorosa en el GI en todos los rangos de edad, especialmente en los niños cuando los PEF son aplicados durante 60 minutos al día 17,23, y otros refieren que el aumento se presenta en ambos géneros 24.

En este estudio existe relación significativa entre el NAF de los escolares y la edad: a menor edad mayor NAF, resultado similar a otros estudios encontrados, en los cuales se afirma que el nivel activo se encuentra relacionado con edades tempranas y con una mayor práctica de AF en la edad adulta 25, es decir, que los comportamientos adquiridos por los escolares perduran durante el curso de la vida 26,27. Por otra parte, otros autores refieren que el NAF post intervención no tiene influencia de esta variable 17,23.

Algunos estudios ponen el énfasis en que las mujeres centran su atención en actividades individuales, de carácter estético, y muestran poco interés por el deporte; además, influyen factores como la vulnerabilidad y la inequidad, mientras que los varones practican actividades de tipo competitivo y colectivas como deportes, lo cual está asociado al aumento del NAF en este sexo 20,28-31, como lo reportan diferentes autores, quienes afirman que las mujeres presentan menor NAF, con diferencias estadísticamente significativas 19,28,32,33. En este estudio estas dos variables no se encuentran relacionadas.

Otras variables sociodemográficas que no influyen en el NAF, según esta investigación, son el estrato socioeconómico y el nivel de escolaridad de la madre; sin embargo, en otros estudios se evidencia que a menor estrato, los escolares son más activos, lo cual se encuentra relacionado con desplazamientos mediante caminata y en bicicleta, además del uso del tiempo libre para jugar con sus pares o ayudar en actividades domésticas 19,25; asimismo, las familias de estos estratos no cuentan con los recursos económicos para acceder a dispositivos tecnológicos, y por tal razón los menores no permanecen la mayor parte del tiempo frente a pantallas, en comparación con niños de estratos altos 20. Con respecto al nivel de escolaridad de la madre, se evidencia que aun cuando cuenten con un nivel de educación superior, que permita tener conocimientos acerca de los beneficios de la práctica de AF, los escolares en mayor proporción fueron inactivos.

En relación con las variables antropométricas, se observaron cambios significativos en el GI entre la pre y la post intervención: el indicador talla/edad ascendió en la valoración final, puesto que los escolares presentaban una talla adecuada para la edad; resultados semejantes refieren otros estudios realizados en adolescentes después de la aplicación de PEF 34. Cabe resaltar que esta variable tiene influencia del factor genético.

Respecto al indicador iMc/edad, no se observaron cambios significativos, aunque hubo una disminución en el número de estudiantes que presentaban sobrepeso y obesidad; resultados similares se observan en otras investigaciones que aplicaron un PEF por 60 minutos, con una intensidad de moderada a vigorosa por 24 semanas, en las cuales el IMC prácticamente no se modificó, disminuyendo levemente al finalizarlo 18,35; a diferencia de otros estudios, se observa una reducción importante del perímetro de cintura e IMC 34 36,37. Un estudio asegura que el IMC en los niños es menor que en las niñas, a causa de lo cual las niñas presentan mayor peso para su edad 34.

En cuanto al perímetro abdominal, en el GI se mantuvo entre la pre y post intervención, en cambio, en el GC aumentó, resultados que se asemejan a los reportados en otros estudios 18,36,38; en contraste, algunos investigadores refieren que no se presentan cambios 39, lo cual puede deberse a la duración de cada sesión, la frecuencia, la intensidad, el tipo de ejercicio y la duración del programa. En este estudio el perímetro abdominal en el GI respecto al NAF fue menor en estudiantes activos, pero sin diferencias significativas respecto del grupo inactivo.

Resultados de diferentes estudios con una duración del programa similar y una frecuencia de 2 veces a la semana evidenciaron mejoras significativas en medidas antropométricas, frecuencia cardíaca, presión sanguínea y triglicéridos 31. En contraste, en este estudio no se reportaron cambios significativos, lo cual es consecuencia de la dificultad para cumplir con la frecuencia recomendada por la OMS; la institución educativa dentro de su currículo solo establece 2 horas semanales de educación física. Además, otras limitaciones fueron la subjetividad para el diligenciamiento del cuestionario PAQ-A, debido a que el escolar puede subestimar o sobrestimar la AF que realiza en los dominios escolar, hogar y tiempo libre 40. De acuerdo con lo anterior, es determinante para obtener resultados significativos hacer uso de instrumentos objetivos, tales como los acelerómetros y los podómetros, los cuales tienen un costo elevado que limita su aplicación.

En conclusión, el NAF activo predomina en el sexo masculino, pero sin diferencias significativas; asimismo, se puede afirmar que decrece con la edad, influido por factores como el tiempo frente a pantallas, actividades extracurriculares, acompañamiento de los padres y acceso a escenarios deportivos. Aunque el PEF no tuvo efectos significativos en las medidas antropométricas de la población objeto de estudio, de forma indirecta se logró sensibilizar a los diferentes actores sobre la importancia de la práctica regular de la actividad física como factor protector de la salud. Entre las recomendaciones sugeridas a la institución educativa se encuentra el promover pausas activas durante la jornada académica, realizar desplazamientos entre cambios de clase y practicar deportes o juego activo en el recreo.

Este programa de ejercicio físico surge como respuesta a las necesidades identificadas en un estudio previo, con el propósito de aumentar el NAF de los escolares, promover estilos de vida saludable, prevenir ENT y generar un impacto positivo en la calidad de vida de niños y adolescentes.
